# Physics-informed neural networks for physiological signal processing and modeling: a narrative review

**DOI:** 10.1088/1361-6579/adf1d3

**Published:** 2025-07-30

**Authors:** Anni Zhao, Davood Fattahi, Xiao Hu

**Affiliations:** Center for Data Science, Nell Hodgson Woodruff School of Nursing, Emory University, Atlanta, GA, United States of America

**Keywords:** physics-informed neural networks, physiological signal processing and modeling, physics models, partial differential equations, ordinary differential equations, forward and inverse problems

## Abstract

Physics-informed neural networks (PINNs) represent a transformative approach to data models by incorporating known physical laws into neural network training, thereby improving model generalizability, reduce data dependency, and enhance interpretability. Like many other fields in engineering and science, the analysis of physiological signals has been influenced by PINNs in recent years. This manuscript provides a comprehensive overview of PINNs from various perspectives in the physiological signal analysis domain. After exploring the literature and screening the search results, more than 40 key studies in the related domain are selected and categorized based on both practically and theoretically significant perspectives, including input data types, applications, physics-informed models, and neural network architectures. While the advantages of PINNs in tackling forward and inverse problems in physiological signal contexts are highlighted, challenges such as noisy inputs, computational complexity, loss function types, and overall model configuration are discussed, providing insights into future research directions and improvements. This work can serve as a guiding resource for researchers exploring PINNs in biomedical and physiological signal processing, paving the way for more precise, data-efficient, and clinically relevant solutions.

## 1. Introduction

Physics-informed neural networks (PINNs) are a class of neural networks that incorporate physical laws directly into the learning process. The idea is to use established scientific principles, often explicitly expressed in ordinary differential equations (ODEs) or partial differential equations (PDEs), as a part of the neural network’s training, guiding the model to solutions that inherently satisfy these physical laws. In conventional neural networks, the model is purely data-driven. Therefore, their performance decreases with limited data or in the presence of noise, distortion, and missing values. PINNs, on the other hand, balance both data and prior knowledge of physics-based constraints. This trade-off allows PINNs to capture complex patterns in data without drastic deviation from the known physics of the phenomena.

The idea of integrating physical prior knowledge into a neural network framework dates back to 1994, when Dissanayake and Phan-Thien introduced a numerical universal approximator, based on neural networks to solve PDEs (Dissanayake *et al* 1994). Afterward, the concept of PINNs has been utilized in different areas with known physics laws and shallow neural networks (Lagaris *et al* 1998, Lagaris *et al* 2000).

Thanks to computing hardware advancements in subsequent decades, the use of deep neural networks with numerous adjustable parameters has become prevalent, which could facilitate leveraging PINNs to solve more detailed and complicated problems (Lai *et al* 2021, Cuomo *et al* 2022). Nowadays, PINNs-based solutions can be seen across various fields of science and engineering, including (but are not limited to) optics (Chen *et al* 2020), electromagnetism (Kovacs *et al* 2022), aerodynamics (Mao *et al* 2020), petroleum engineering (Abreu *et al* 2021), etc.

**Figure 1.**
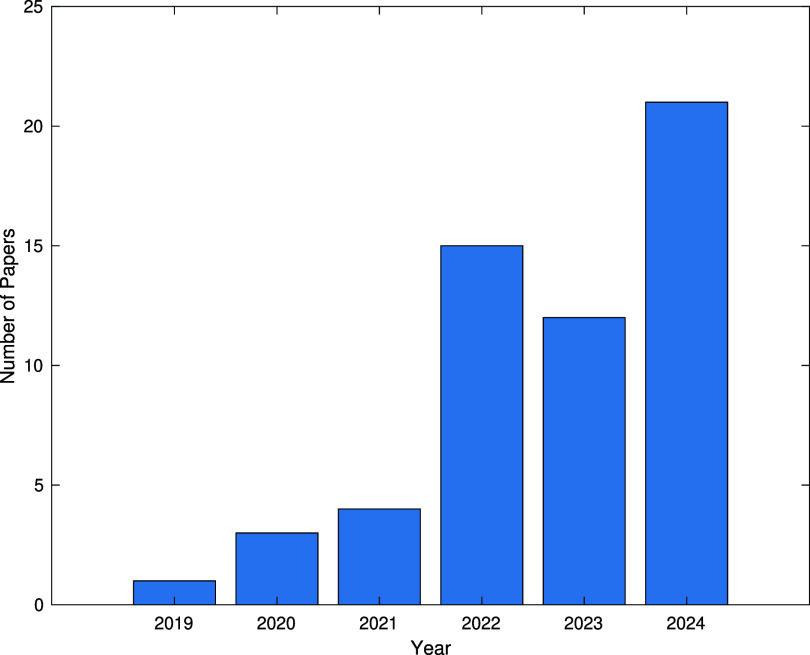
Number of published PINNs studies in physiological signal processing and modeling.

**Figure 2.**
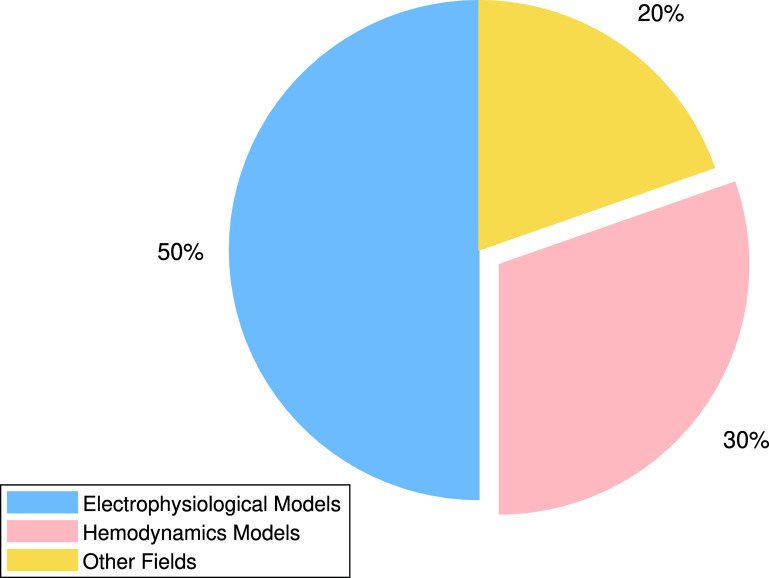
Percentage of published PINNs studies in each field of physiological signal processing and modeling.

In medicine, the analysis of physiological data presents some of the most suitable problems for modeling with ODEs/PDEs and solving with PINNs. Figure 1 shows the growing trend of publications in this field in recent years, serving as an indicator of the increasing importance and potential of this method. Among the various types of problems, the study of electrical signals propagation (electrophysiology) and blood flow in cardiovascular system (hemodynamics) are the two most remarkable fields in the literature in which PINNs are deployed, as shown in figure 2.

However, to the best of our knowledge, a comprehensive review of PINNs methods, particularly their applications in solving physiological and biomedical problems, is lacking in the literature. While individual studies have explored specific implementations, a review that systematically categorizes their methodologies, governing ODEs/PDEs, neural network architectures, and objectives across different domains remains largely unexplored.

In this study, we review the literature on the application of PINNs in biomedical and physiological data analysis from April 2019 to December 2024. The selected papers were sourced from various databases and digital libraries, including Google Scholar, PubMed, and IEEE Xplore, using a combination of targeted keywords to maximize the retrieval of relevant studies. The keywords used in the various research engines are listed as follows:

*Keyword 1: ‘Physics informed neural networks’ AND ‘Physiological signal modeling’*

*Keyword 2: ‘Hybrid modeling’ AND ‘Neural networks’ AND ‘Physiological signal modeling’*

The following is a sample query utilized to filter the search results:

*(Physics-informed $|$ Theory-Trained $|$ customized $|$ prior information $|$ Bayesian $|$ function approximation $|$ differential equation) & (neural networks) & (Electrophysiology $|$ Hemodynamics $|$ physiological signals)*

**Figure 3.**
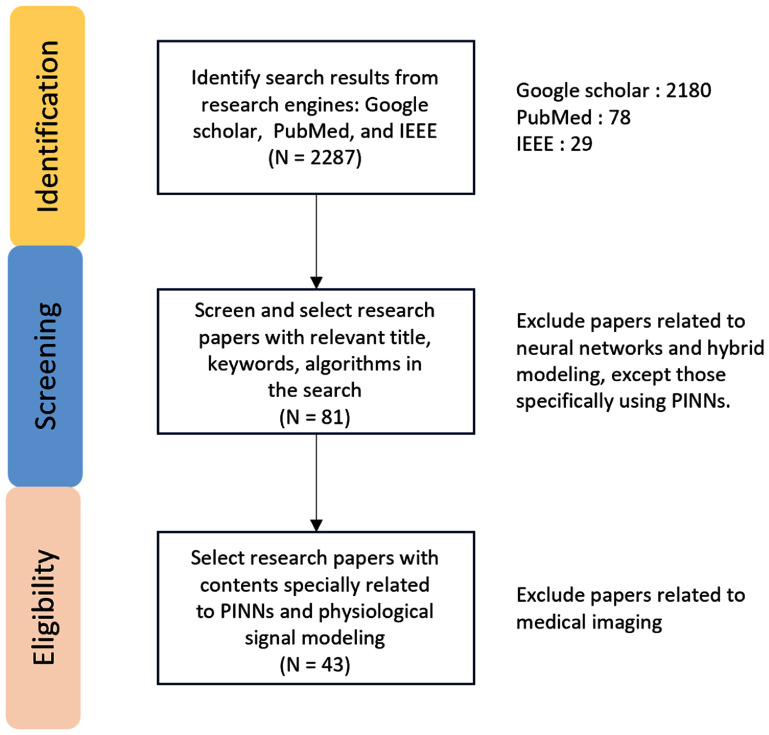
The search methodology adopted in this paper to locate suitable references.

A flowchart is shown in figure 3 to demonstrate the search strategy for this paper. Articles on biomedical imaging are excluded to maintain focus on studies utilizing PINNs for physiological signal processing and modeling. Additional characteristics, including the application fields, ODEs/PDEs properties, and neural network architectures, are identified by manual inspection and detailed analysis of the articles.

The next sections are organized as follows: section 2 explores the architecture of PINNs framework, structure of embedded neural networks, and the associated loss functions. Section 3 describes the prevalent PDEs and ODEs used in the application of PINNs to physiological signal processing and modeling. Section 4 summarizes the objectives, key characteristics, and performance metrics of PINNs in various areas. In section 5, the general aims and findings of the study are discussed. Finally, section 6 concludes the paper.

## 2. Framework of PINNs in physiological signal

### 2.1. Neural networks structure

In this section, the architecture and framework of PINNs in physiological signal processing and modeling are explained in detail. PINNs leverage the universal function approximation property of neural networks to incorporate multiple physical constraints into the loss function (Raissi *et al* 2019, Cuomo *et al* 2022). Machine learning has been extensively adopted in areas such as image recognition, natural language processing, and autonomous systems by leveraging supervised learning, unsupervised learning, and reinforcement learning techniques. The application of machine learning has also been seen in the area of scientific computing. By incorporating physical laws into the neural networks learning process, PINNs are recognized as powerful tools for solving ODEs/PDEs across various fields, including computational fluid dynamics (CFD) (Raissi *et al* 2020), heat transfer (Raissi *et al* 2019), and structural mechanics (Rao *et al* 2021) without labeled data. For physiological signal analysis and modeling, PINNs are utilized to address both forward and inverse problems (Li *et al* 2024). PINNs serve as solvers for ODEs/PDEs with given parameters, boundary conditions, and initial conditions in forward problems. On the other hand, in inverse problems, PINNs serve as parameter estimators to determine unknown parameters within a system governed by ODEs/PDEs using observed data. Various neural network structures have been adopted in PINNs for physiological signal processing and modeling, such as convolutional neural network (CNN) (Jiang *et al* 2024), residual network (ResNet) (Xie *et al* 2023), autoencoder (Tenderini *et al* 2022), and Fourier-based activation function (Aghaee and Khan 2024), etc. Table 1 provides a detailed explanation of the representative architecture and framework of PINNs in terms of application areas, type of problems, and neural networks structures.

**Table 1.** Type of application areas, problems, and neural networks structures of PINNs in physiological modeling.

Area	Type	Neural networks structures
Cardiac electrophysiology & Hemodynamics	Forward	**Feed-forward neural networks**
		Sahli *et al* (2020)
		Xie and Yao *et al* (2022b)
		Herrero *et al* (2022)
		Maidu *et al* (2025)
		Kissas *et al* (2020)
		**CNN**
		Jiang *et al* (2024)
		Nazaret *et al* (2023)
		**DeepONet**
		Li *et al* (2024)
		**Fourier-based activation function**
		Aghaee and Khan (2024)
		**Neural network finite element model**
		Motiwale *et al* (2024)
		Zhang *et al* (2022)
	Inverse	**Autoencoder**
		Tenderini *et al* (2022)
		Nazaret *et al* (2023)
	Forward & Inverse	**RNN**
		Xie (2023)
		Kashtanova *et al* (2022b)
		Tenderini *et al* (2022)
		Jiang *et al* (2024)

Neural dynamics	Forward	**Transformer networks**
		Sarabian *et al* (2022)
	Inverse	**Adversarial contrastive learning**
		Wang *et al* (2024)

Cancer	Forward	**LSTM, U-Net**
		Ottens *et al* (2022)
		**Feed-forward neural networks**
		Mukhmetov *et al* (2023)

Electromyography	Forward	**CNN**
		Li *et al* (2022)
		**Feed-forward neural networks**
		Taneja *et al* (2022)
		Ma *et al* (2024)
		Zhang *et al* (2022)

PINNs have been extensively utilized in cardiac electrophysiological (EP) modeling (Herrero *et al* 2022, Kashtanova *et al* 2022b, Chiu *et al* 2024) and in addressing cardiovascular blood flow problems (Liang *et al* 2023, Aghaee and Khan 2024, Bhaumik *et al* 2024, Li *et al* 2024). In most cases, PINNs are employed to solve forward problems involving the Navier–Stokes equation (Li *et al* 2024), the Eikonal equation (Sahli *et al* 2020, Jiang *et al* 2024), the Aliev–Panfilov model (Herrero *et al* 2022, Tenderini *et al* 2022, Xie and Yao 2022a), and various other cardiac electrodynamics models (Nazaret *et al* 2023, Chiu *et al* 2024, Jiang *et al* 2024). Details of these physiological models are introduced in section 3. Gerogios (Kissas *et al* 2020) introduced a PINNs framework to model cardiovascular flows and predict arterial blood pressure with clinically acquired non-invasive MRI data using general feed-forward neural networks. The proposed neural networks structure has been analyzed and shown to possess sufficient capacity to efficiently capture fine details in propagating waveforms. A separate PINNs framework for cardiac activation mapping was proposed by Sahli Costabal *et al* (2020), also employing a feed-forward neural network structure. Results from benchmark problems show that the PINNs method outperforms linear interpolation and Gaussian process regression with fewer samples. Bahetihazi (Maidu *et al* 2025) explores the use of traditional PINNs with momentum balance derived from the Navier–Stokes equation to enhance the resolution of intraventricular vector flow mapping without using color-Doppler input data. In this study, PINNs demonstrate excellent performance in terms of aliasing artifacts and recovering missing velocity values. A new physics-informed neural operator to compute the red blood blow continuously both with location and time using the Navier–Stokes equation is developed in Li *et al* (2024). Additionally, Kuang *et al* (2024) proposed a physics-informed self-supervised learning model to identify digital twins of cardiac hemodynamics using noninvasive echocardiogram videos. A framework named augment incomplete PHYsical models with a deep learning component for ideNtifying complex cardiac ElectroPhysiology dynamics (APHYN-EP) (Kashtanova *et al* ) was introduced to correct and learn cardiac electrophysiology dynamics based on a low-fidelity physical model. A deep learning structure is adopted to model the information that cannot be captured by the physical model. The 3D Navier–Stokes equation, combined with PINNs, is adopted to inversely estimate the blood flow parameters using feed-forward neural networks (Iasev *et al* ). The generation of the potential energy function was personalized in a previous study (Buoso *et al* 2021). Additionally, PINNs have been applied to model cardiac electrophysiology particularly in the context of fibrillation (Chiu *et al* 2024), and to perform both forward and inverse analysis in electrocardiographic imaging (Jiang *et al* 2024).

Limited applications of PINNs have also been explored in areas such as brain hemodynamics, neural dynamics, cancer modeling, and other specialized domains. An innovative approach was introduced in Sarabian *et al* (2022) by combining transformer networks with a one-dimensional reduced-order model to estimate brain hemodynamic parameters. Furthermore, an adversarial contrastive learning-based PINNs algorithm was developed by Wang *et al* (2024) to estimate the blood pressure cufflessly. PINNs have also been applied to the thermal modeling of patient-specific breast cancer (Perez-Raya and Kandlikar 2023) and for parameter estimation in pancreatic cancer using DCE-MRI data (Ottens *et al* 2022). In Ottens *et al* (2022), the fully-connected network (FCN), long short-term memory neural network (LSTM), and gated recurrent unit (GRU) are adopted to construct temporal frameworks. U-Nets have been adopted to investigate the performance of two spatio-temporal frameworks. In general, feed-forward neural networks have been extensively adopted in physiological signal processing and modeling with certain accuracy and efficiency. Forward prediction results can be further improved by using advanced neural networks structures such as CNNs, DeepONet, and physics-informed neural operators, etc. Autoencoder structures are frequently applied in inverse problems for parameter estimation. Owning to the rapid development of machine learning, numerous advanced neural network architectures have been developed. The choice of neural network structure for PINNs depends on the specific problem being addressed.

### 2.2. Loss function

In addition to various neural network structures, multiple physics constraints and loss functions are also adopted and designed to model physiological systems in PINNs. The loss function is a critical part in the design of PINNs by incorporating the data-driven and physics-based constraints. Here, a general form of the loss function for PINNs is given in equation (1),

\begin{equation*} \mathcal{L}_{\text{total}} = \lambda_1\mathcal{L}_\mathrm {Physics} + \lambda_2\mathcal{L}_\mathrm {BC} + \lambda_3\mathcal{L}_\mathrm {IC} + \lambda_4\mathcal{L}_\mathrm {Data} + \lambda_5\mathcal{L}_\mathrm {Reg} + \lambda_6\mathcal{L}_\mathrm {Interface}\end{equation*}

where

• $\mathcal{L}_\mathrm {Physics}$ is the loss for the physics-based governing differential equations.
• $\mathcal{L}_\mathrm {BC}$ is the loss for the boundary conditions of the governing differential equations.
• $\mathcal{L}_\mathrm {IC}$ is the loss for the initial conditions of the governing differential equations.
• $\mathcal{L}_\mathrm {Data}$ is the loss for the measurement data.
• $\mathcal{L}_\mathrm {Reg}$ is the loss for the regularization term.
• $\mathcal{L}_\mathrm {Interface}$ is the loss for the interface points at bifurcations or junctions.
• *λ*_*i*_, $i = 1,\cdots,6$ are the weighting factors.

The mean squared error (MSE) or averaged mean squared error (AMSE) is generally adopted to construct the loss function for evaluating and optimizing PINNs. The physics-based governing differential equations, the boundary conditions, the initial conditions, and the measurement data serve as typical physics constraints in PINNs. All the loss terms in PINNs are formulated based on the outputs of the neural networks. In particular, $\mathcal{L}_\mathrm {Physics}$ is the loss term derived from physics-based ODEs/PDEs, while $\mathcal{L}_\mathrm {BC}$, $\mathcal{L}_\mathrm {IC}$, $\mathcal{L}_\mathrm {Data}$, and $\mathcal{L}_\mathrm {Interface}$ are the loss terms formulated based on boundary conditions, initial conditions, data constraints, and interface constraints. Furthermore, a regularization term, $\mathcal{L}_\mathrm {Reg}$, is employed in Tenderini *et al* (2022) and Sahli Costabal *et al* (2020) to help solve ill-posed inverse problems and prevent data overfitting. Weighting factors are included in the loss function to adjust the contribution of each loss term. In most cases, the weighting factors are typically set to one. The weighting parameters *λ*_*i*_ are used to balance the losses associated with measurement data, boundary conditions, and PDEs, as demonstrated in Xie and Yao (2022b), Wang *et al* (2024), Maidu *et al* (2025). The weighting factor can also be adjusted dynamically during training to modify the contribution of individual loss terms (Kashtanova *et al* 2022b). Additionally, contrastive learning loss has been innovatively combined with adversarial sample loss to enhance the model training process (Wang *et al* 2024). Automatic differentiation (Baydin *et al* 2018) plays a pivotal role in the development and implementation of PINNs. By allowing exact and efficient computation of derivatives, automatic differentiation makes it possible to embed the governing ODEs/PDEs into the loss function—in $\mathcal{L}_\mathrm {Physics}$, $\mathcal{L}_\mathrm {BC}$, and $\mathcal{L}_\mathrm {IC}$ — thereby ensuring that the neural network outputs adhere to the underlying physical laws.

Here we adopt a PDE as an example to further illustrate the formulation of the loss terms. Consider the non-homogeneous PDE given as,

\begin{align*} \mathcal{N}[u(x,t)] &amp; = f(x,t), (x, t) \in \Omega \times (0, T]\end{align*}

\begin{align*} u\left(x,0\right) &amp; = u_0\left(x\right), x\in \Omega.\end{align*}

The boundary condition is given as,

\begin{equation*} \mathcal{B}[u(x,t)] = 0, (x,t)\in\partial\Omega\times(0, T].\end{equation*}

Let $\Omega\subset\mathbb{R}^n$ be a spatial domain with boundary $\partial \Omega$, and let $t\in[0,T]$ denotes the temporal interval. *x* is defined as the spatial variable, $u(x,t)$ is the solution of the PDE. $u_0(x)$ is defined as the initial condition. $\mathcal{N}[\cdot]$ is defined as the differential operator, $\mathcal{B}[\cdot]$ is defined as the boundary operator. In PINNs framework, the solution of PDE is approximated by the neural networks as $\hat{u}_{\theta}(x,t)$, then the format of the loss terms in equation (1) can be defined as,

\begin{align*} \mathcal{L}_\mathrm {Physics} &amp; = \frac{1}{N_f}\sum_{i = 1}^{N_f}\left(\mathcal{N}\left[\hat{u}_\theta\left(x_p^i,t_p^i\right)\right] - f\left(x_p^i, t_p^i\right)\right)^2\end{align*}

\begin{align*} \mathcal{L}_\mathrm {BC} &amp; = \frac{1}{N_b}\sum_{i = 1}^{N_b}\left(\mathcal{B}\left[\hat{u}_\theta\left(x_b^i,t_b^i\right)\right]\right)^2\end{align*}

\begin{align*} \mathcal{L}_\mathrm {IC} &amp; = \frac{1}{N_0}\sum_{i = 1}^{N_0}\left(\hat{u}_\theta\left(x_{ic}^i,0\right)-u_0\left(x_{ic}\right)\right)^2\end{align*}

\begin{align*} \mathcal{L}_\mathrm {Data} &amp; = \frac{1}{N_u}\sum_{i = 1}^{N_u}\left(\hat{u}_\theta\left(x_\mathrm {data}^i, t_\mathrm {data}^i\right) - u\left(x_\mathrm {data}^i, t_\mathrm {data}^i\right)\right)^2\end{align*}

\begin{align*} \mathcal{L}_\mathrm {Reg} &amp; = \frac{\lambda_{reg}}{2}\sum_{j = 1}^{P}\theta_j^2\end{align*}

\begin{align*} \mathcal{L}_\mathrm {Interface} &amp; = \frac{1}{N_\mathrm {int}}\sum_{i = 1}^{N_\mathrm {int}}\left(\hat{u}_\theta\left(x_{if}^i, t_{if}^i\right) - u\left(x_{if}^i, t_{if}^i\right)\right)^2\end{align*}

where *N_f_* is the number of collocation points for the PDE residuals, $x_p^i$ represents the spatial variables at which the PDE residual is evaluated, $t_{p}^i$ denotes the corresponding time values. *N_b_* is the number of points located at the boundary conditions, $x_b^i$ represents the spatial variables located at the boundary conditions, $t_{b}^i$ denotes the time variables at which boundary conditions are enforced. *N*_0_ is the number of points for the initial conditions, $x_{ic}^i$ represents the spatial variables at initial time *t* = 0. *N_u_* is the number of points for the observable data, $x_\mathrm {data}^i$ represents the spatial variables at the locations where measured data are available, $t_\mathrm {data}^i$ denotes the time points corresponding to those measurements. *θ*_*j*_ is the $j\textrm{th}$ parameter in the neural networks, P is the total number of trainable parameters, $\lambda_\mathrm {reg}$ is the regularization coefficient. $N_\mathrm {int}$ is the number of points at the interface, $x_{if}^i$ represents the points at an interface between domains, $t_{if}^i$ denotes the time points at which interface continuity is located. Here, we use *L*_2_ regularization to illustrate the regularization loss term $L_\mathrm {Reg}$, and adopt MSE as an example to demonstrate the formulation of the loss terms.

### 2.3. Overview of PINNs frameworks

**Figure 4.**
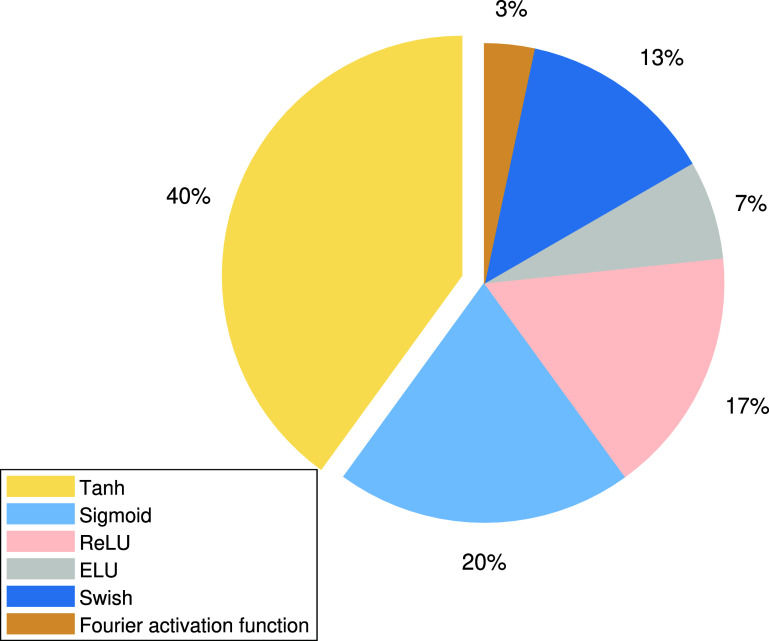
Distribution of the activation functions for PINNs reviewed from 30 articles in physiological signal processing and modeling.

**Figure 5.**
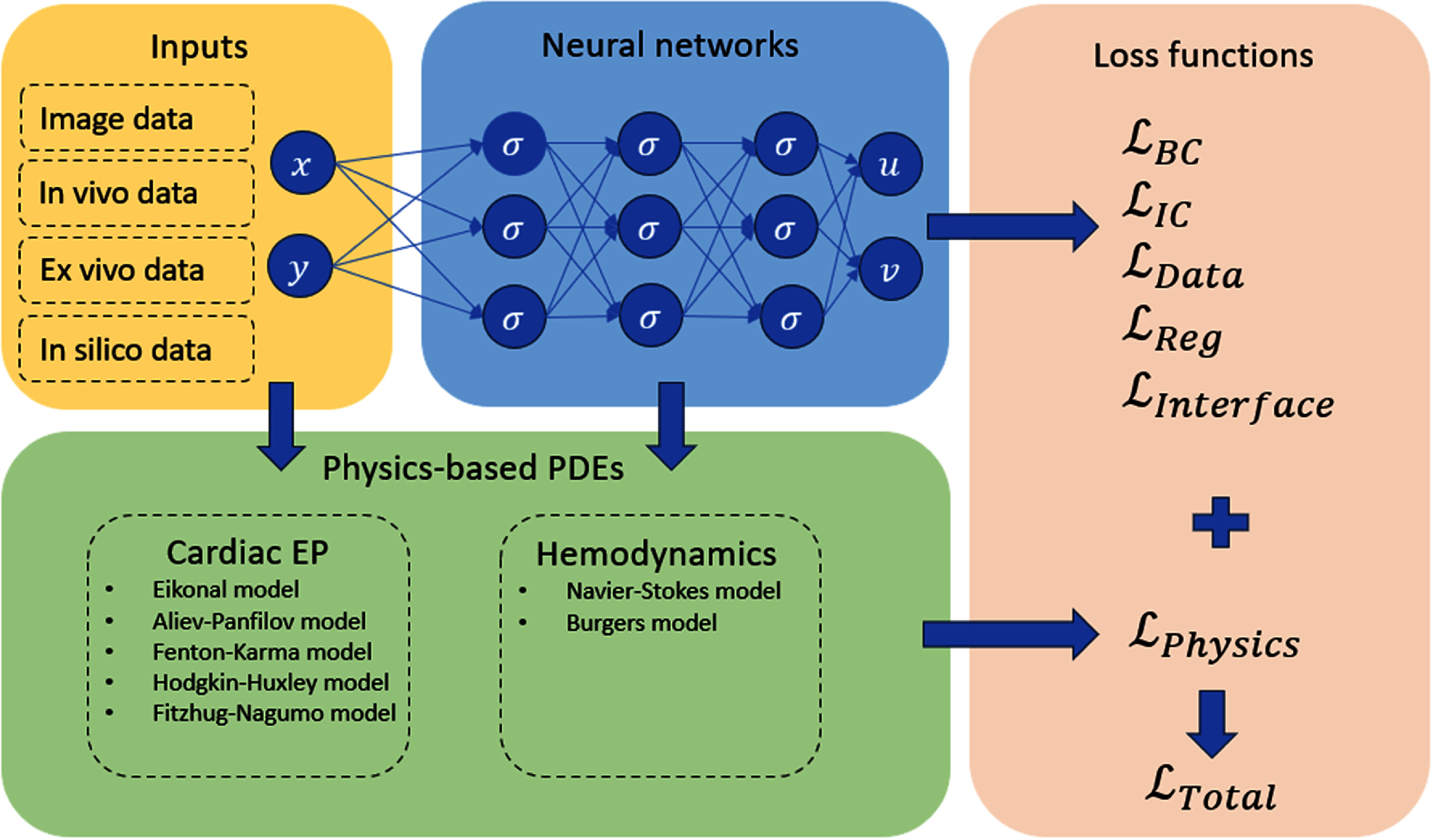
Schematic illustration of the PINNs framework, including inputs, neural networks, physics-based PDEs, and loss functions.

Physics models, neural network structures, loss functions, pre-processing of input data, and activation functions are all critical components of physiological modeling using PINNs. Physiological modeling using PINNs adopts multiple input data modalities, including images and signals, often derived from *in vivo*, *ex vivo*, or *in silico* sources. Non-dimensional and normalization are performed on the network inputs and have been shown to enhance the performance of PINNs (Kissas *et al* 2020, Du *et al* 2023). Although the activation function is not the most critical factor in the performance of PINNs, its selection remains important to guarantee the reliability of the results. The distribution of activation function for PINNs, as reviewed in 30 articles, is shown in figure 4. Hyperbolic tangent, sigmoid, and rectified linear unit (ReLU) are the most popular activation functions in PINNs, collectively appearing in 77% of the review articles. Swish and exponential-linear unit (ELU) are also widely adopted, accounting for 20% among the articles. The Fourier activation function is a special activation function by making use of the Fourier series. It has been adopted in Aghaee and Khan (2024) to enhance the ability of neural networks to model periodic or oscillatory functions. In figure 5, the structures of physics models, inputs, neural networks, and loss functions are summarized. Here, the ODEs/PDEs used in cardiac electrophysiology and brain hemodynamics are adopted as examples. To formulate a PINNs framework, there are several key components, including the PDE with initial and boundary conditions, measurement data, neural network structure, loss function definition, and optimizer chosen. The governing physics law is fitted into the neural network with measurement data simultaneously to train the neural networks, using automatic differentiation to compute the PDE residuals, boundary condition residuals, and initial condition residuals as part of the loss function. Table 2 shows a summary for the input data types, evaluation metrics, training time, and prevalent optimizers in PINNs. *In silico* data is commonly used to validate PINNs, while *in vivo*, *in vitro*, and *ex vivo* are also utilized during model development. *In silico* data is generated noise free using fully developed mathematical models or simulations, which plays an important role in the development and validation of PINNs framework before transitioning to clinical applications. *In vivo*, *in vitro*, and *ex vivo* are always noisy and sparse, making initial model testing and training more challenging. Evaluation metrics typically include mean squared error (MSE), averaged mean squared error(AMSE), root mean squared error (RMSE), and mean absolute error (MAE), with additional metrics like mean absolute percent error (MAPE), normalized RMSE (NRMSE), and relative error (RE) are used occasionally. Training time ranges from 131.29 s to 110 h, depending on the complexity of the model, hardware setup, and the optimizers employed. Popular optimizers include Adam (Adaptive Moment Estimation), L-BFGS (Limited-memory Broyden-Fletcher-Goldfarb-Shanno), and Nadam (Nesterov-accelerated Adaptive Moment Estimation). Parallel computing can also be considered in PINNs to enhance computational efficiency by leveraging the computing power of multiple processors. To provide clearer guidance to the reader for modeling PINNs in the future, table 3 presents a summary of data availability and open-source code resources.

**Table 2.** Summary for the input data types, evaluation metrics, training time, and optimizers in PINNs.

Characteristics	Details
Data type	*In silico* data
	*In vivo* data
	*In vitro* data
	*Ex vivo* data

Evaluation metrics	MSE (Mean squared error)
	AMSE (Averaged mean squared error)
	RMSE (Root mean squared error)
	MAE (Mean absolute error)
	MAPE (Mean absolute percent error) (Nazaret *et al* 2023)
	NRMSE (Normalized root mean squared error)
	RE (Relative error) (Xie and Yao 2022a, Isaev *et al* 2024a)
	ME (Mean error)

Training time	$131.29\,\mathrm{s} \sim110\,\mathrm{h}$

Optimizer	Adam
	L-BFGS (Herrero *et al* 2022)
	Nadam (Tenderini *et al* 2022)

**Table 3.** Summary of the data availability and open-source code resources.

Data/Source code	References
Data	MIMIC datasets (Li *et al* 2024)
	Arterial blood flow datasets (Bhaumik *et al* 2024)
	Cardiac electrophysiological datasets (Kashtanova *et al* 2022a)
	Cardiovascular flows datasets (Kissas *et al* 2020)
	Cuffless blood pressure datasets (Wang *et al* 2024)
	EEG datasets (Sun *et al* 2022)

Open-source code	Aghaee and Khan (2024)
	Sahli *et al* (2020)
	Herrero *et al* (2022)
	Kissas *et al* (2020)
	Nazaret *et al* (2023)
	Wang *et al* (2024)
	Ottens *et al* (2022)
	Bhaumik *et al* (2024)
	Kashtanova *et al* (2022a)
	Alzhanov *et al* (2024)
	Sun *et al* (2022)

## 3. Physics models and prior information

In this section, the physics models and prior information adopted in PINNs for physiological signal processing and modeling are explained in detail. In addition, the residual definitions of the loss functions for various models are introduced in detail. This section covers EP models, muscle electromechanical models, and hemodynamic models.

### 3.1. Electrophysiological models

The properties of cells’ electrical activities have long been indicators of an organ’s health or dysfunction. For example, studies indicate that areas of the myocardium with abnormal EP properties, such as reduced conduction velocity, increased excitability, or shortened action potential duration (APD), are more likely the triggering sources for arrhythmias like atrial fibrillation (AF) (Nattel *et al* 2017).

The pattern of electrical signals generation and propagation across myocardium cells can be modeled mathematically by PDEs (Clayton *et al* 2011). This is the first key factor in order to simulate the cardiac EP behavior using PINNs. Another important factor is the volume boundary condition to prevent electrical signal leakage outside the heart domain, which can also be mathematically modeled. Therefore, with appropriately formulated PDEs and boundary conditions, coupled with a neural network structured with sufficient layers, it becomes feasible to construct PINNs that can effectively tackle both forward and inverse problems. In the following, several well-known models for analyzing EP signals using PINNs are discussed.

#### 3.1.1. Eikonal model

The Eikonal PDE is the simplest model in cardiac EP activities analysis. It describes the propagation of electrical activity wavefront across the heart using principles derived from geometric optics. In this context, the wavefront is treated somewhat like a light wave, where the speed of propagation and the direction are governed by the local properties of the tissue. The equation is given by,

\begin{align*} D\left(x\right) \nabla \phi\left(x\right) \cdot \nabla \phi\left(x\right) = 1\end{align*}

where $\phi(x) $ represents the activation time at a location *x*, and *D*(*x*) is the conductivity tensor.

The Eikonal model has been frequently used as the PDE in PINNs for EP analysis. By leveraging the Eikonal model, the PINNs framework can enforce physically realistic constraints on the propagation of these signals, improving both the accuracy and the clinical relevance of the activation maps (Sahli *et al* 2020).

Sometimes the integration of the Eikonal model as part of a broader set of models in PINNs framework can enhance the personalization of cardiac electrophysiology simulations (Jiang *et al* 2024). The model parameters can also be adaptively adjusted using meta-learning techniques, and merging physics-informed methods with adaptive neural network capabilities (Jiang *et al* 2024).

The Eikonal model can also be employed to enhance the understanding of atrial fiber orientations and conductivity tensors. The model helps in interpreting complex intracardiac electrograms by providing a physics-informed structure to the learning process, ensuring that the predictions are consistent with known physiological behaviors (Grandits *et al* 2021).

#### 3.1.2. State-space model (SSM)

State-space modeling provides a framework to express the current state of electrical activation in terms of its past states. When combined with neural networks, a hybrid neural SSM can represent the mapping of cardiac electrical activity at each point on the heart to body surface measurements. Accordingly,

\begin{align*} \frac{\mathrm ds_t}{\mathrm dt} &amp; = F_\phi\left(s_t\right)\end{align*}

\begin{align*} X_t &amp; = D_\eta\left(s_t\right)\end{align*}

where the first equation governs temporal evolution of latent state at time *t* through neural network *F*_*φ*_, and the spatial decoder *D*_*η*_ maps latent state to cardiac potentials *X_t_*, *s_t_* is the state variable. Finally, the observed body-surface ECG measurements at time *t* can be obtained by a physics-based emission model,

\begin{align*} Y_t = H X_t\end{align*}

where *H* is the lead-field matrix (a linear operator from quasi-static electromagnetic field theory). This structure defines a neural SSM where the temporal component is learned via neural ODEs and the spatial structure via graph CNNs (GCNNs). The training objective combines a physics-based loss to ensure $Y_t \approx H X_t$, and a data-driven loss to penalize prediction error when ground truth *X_t_* is available. Employing the SSM in PINNs enables a hybrid neural state-space approach to reconstruct electrocardiographic images. This leads to improved spatial resolution and accuracy in ECG imaging (ECGI), which is pivotal for identifying regions of cardiac tissue that may be responsible for arrhythmias (Jiang *et al* 2024).

#### 3.1.3. Aliev–Panfilov model

Among various PDEs, the Aliev–Panfilov model and its variations are the most prevalent ones to model the EP activities of myocardium. The model’s simplicity and nonlinear nature make it a powerful tool for simulating complex phenomena in cardiac electrophysiology. They usually include two terms changing across the volume by time; the first component, simulated by a PDE, represents the electrical diffusion and excitation, and the second component is the relaxation and recovery of the tissue which is modeled by an ODE (Aliev and Panfilov 1996),

\begin{align*} \frac{\partial V}{\partial t} = E \nabla\left(D_E \nabla V\right) - kV\left(V - a\right)\left(V - 1\right) - VW\end{align*}

\begin{align*} \frac{\mathrm dW}{\mathrm dt} = \left( \epsilon + \frac{\mu_1 W}{V + \mu_2} \right) \left( -W - kV\left(V - b - 1\right) \right)\end{align*}

where in the first equation *V* is the transmembrane voltage to describe the electrical potential across the cell membrane, *t* is the time, $\nabla$ is the divergence operator used for spatial diffusion, *D_E_* is diffusion coefficient or conductivity tensor, *E* is anisotropy or fiber direction modifier, which often is a normalized direction vector, *k* is constant related to reaction kinetics or ion channel dynamics, *a* is the threshold potential for activation, and *W* is a recovery variable related to tissue refractoriness or recovery. In the recovery equation *W* is the recovery variable that models the repolarization or refractoriness of tissue, *ε* is a small constant controlling recovery dynamics baseline, *µ*_1_ is the scaling parameter for nonlinearity in *W* dynamics, and *b* is the parameter defining the shape of the recovery function.

The Aliev–Panfilov model has been widely used in PINNs-based cardiac electrophysiology research, including applications such as adaptive learning and electrocardiographic imaging. However, in most studies, it is not embedded as the governing PDE within the PINNs framework. Instead, its primary role is to generate synthetic datasets for training, validation, or evaluation of other models (such as the Eikonal model) within PINNs (Tenderini *et al* 2022, Jiang *et al* 2024).

The successful use of the Aliev–Panfilov model as a governing PDE directly within the PINNs framework has been reported in two recent studies. In the first one, it is incorporated into a PINNs framework for spatiotemporal modeling of cardiac activity, enabling the estimation of heart electrical signals from the intra-cardiac signals, enhancing learning process from sparsely sampled data, and improving model predictions (Xie and Yao 2022a). The second study employs a similar framework but for heart surface potential mapping estimation from surface ECG (ECGI inverse problem) (Xie and Yao 2022b).

In another study, the Aliev–Panfilov model serves as the basis for formulating the PINNs’ physics-based constraints, allowing them to solve forward problems (e.g. action potential propagation) and inverse problems (e.g. parameter estimation) in a simplified setting of 1D and 2D spaces. The model demonstrates high accuracy in characterizing tissue properties, even in the presence of heterogeneities like fibrosis (Herrero *et al* 2022). In a more recent and comprehensive study, the Aliev–Panfilov model is employed to simulate wave propagation under various conditions, including sinus rhythm, tachycardia, and fibrillation in both 2D and 3D geometries (Chiu *et al* 2024). The paper demonstrates the scalability of PINNs in handling complex geometries and dynamic conditions.

#### 3.1.4. Fenton–Karma model

Another model used for cardiac EP simulation is the Fenton–Karma model. Similar to Aliev–Panfilov, this model also simulates the diffusion and recovery phases, but with higher degrees of freedom, complexity and nonlinearity. It typically involves three variables representing the membrane potential, a fast recovery variable, and sometimes a slow recovery variable. One PDE plus two ODEs simulate dynamics of these three variables, which allow to model different phases of the cardiac action potential with a reasonable degree of complexity (Fenton and Karma 1998),

\begin{align*} \frac{\partial u}{\partial t} &amp; = D \nabla^2 u - \left( J_{\text{fi}}\left(u, v\right) + J_{\text{so}}\left(u\right) + J_{\text{si}}\left(u, w\right) \right)\end{align*}

\begin{align*} \frac{\mathrm dv}{\mathrm dt} &amp; = \begin{cases} \frac{1 - v}{\tau_v^-}, &amp; \text{if } u < u_c \\ - \frac{v}{\tau_v^+}, &amp; \text{if } u \unicode{x2A7E} u_c \end{cases}\end{align*}

\begin{align*} \frac{\mathrm dw}{\mathrm dt} &amp; = \begin{cases} \frac{1 - w}{\tau_w^-}, &amp; \text{if } u < u_c \\ - \frac{w}{\tau_w^+}, &amp; \text{if } u \unicode{x2A7E} u_c \end{cases}\end{align*}

where *u* is the normalized transmembrane potential (dimensionless, typically scaled to [0, 1]), *v* is the gating variable for fast inward current *J*_fi_ (Na^+^ inactivation), *w* is the gating variable for slow inward current *J*_si_ (Ca^2+^ inactivation), *D* is the diffusion coefficient (mm^2^ ms^−1^), representing isotropic conductivity, $\nabla^2 u $ is the Laplacian of the potential, modeling spatial diffusion, $J_{\text{fi}}(u, v) $ is the fast inward current (analogous to Na^+^ influx), $J_{\text{so}}(u) $ is the slow outward current (analogous to K^+^ efflux), $J_{\text{si}}(u, w) $ is the slow inward current (analogous to Ca^2+^ influx), $\tau_v^-$ and $\tau_v^+$ are the time constants controlling the recovery/inactivation of *v*, $\tau_w^-$ and $\tau_w^+$ are the time constants controlling the recovery/inactivation of *w*, *u_c_* is voltage threshold that governs switching behavior of *v* and *w*.

PINNs based on the Fenton–Karma model are rare in the literature, but it usually is employed as a simulation tool to generate synthetic ground truth data. It is not directly embedded into the PINNs frameworks but is instrumental in evaluating their performance against highly detailed EP simulations (Sahli *et al* 2020, Chiu *et al* 2024).

#### 3.1.5. Mitchell–Schaeffer model

The Mitchell–Schaeffer model is a simplified mathematical model used to simulate the EP behavior of cardiac cells. It focuses on the essential dynamics of action potential propagation while minimizing computational complexity, making it suitable for large-scale simulations and integration with machine learning frameworks like PINNs (Ayed *et al* 2019, Kashtanova *et al* 2021, Kashtanova *et al* 2022a). Unlike detailed models (e.g. Hodgkin–Huxley or Fenton–Karma), the Mitchell–Schaeffer model abstracts ionic currents into simplified terms, focusing on the essential dynamics of excitation and recovery. The Mitchell–Schaeffer model consists of 1 PDE for the transmembrane potential *v*, and 1 PDE for the gating variable *h* given as,

\begin{align*} \frac{\partial v}{\partial t} = \nabla \cdot \left(\sigma I \nabla v\right) + \frac{h v^2 \left(1-v\right)}{\tau_{\text{in}}} - \frac{v}{\tau_{\text{out}}} + J_{\text{stim}}\end{align*}

\begin{align*} \frac{\partial h}{\partial t} = \begin{cases} \frac{1-h}{\tau_{\text{open}}}, &amp; \text{if } v < v_{\text{gate}} \\ -\frac{h}{\tau_{\text{close}}}, &amp; \text{if } v > v_{\text{gate}} \end{cases}\end{align*}

where *v* is the transmembrane potential (dimensionless variable representing electrical activity), *h* is the gating variable that controls the recovery (repolarization) dynamics, *σ* is the conductivity scalar (or tensor, in general), *I* is the identity matrix, used in diffusion modeling, $\tau_{\text{in}} $ is the time constant for inward depolarizing current, $\tau_{\text{out}} $ is time constant for repolarization, $\tau_{\text{open}} $ is the time constant for opening the gate variable *h*, $\tau_{\text{close}} $ is the time constant for closing the gate variable *h*, *v*_gate_ is the threshold value of *v* to determine the gating regime, *J*_stim_ is the external stimulus current.

The first PDE captures the spatiotemporal propagation of electrical activity in cardiac tissue and the second PDE describes the temporal evolution of the gating variable that influences tissue refractoriness.

Two recent studies employ the Mitchell–Schaeffer model as the physical framework for their deep learning approaches. The equations governing the model are identical, with the focus on the transmembrane potential and the gating variable. The PINNs are designed to solve both forward and inverse problems, learning the dynamics from data simulated with the Mitchell–Schaeffer equations while addressing variable conditions like scar geometries and conduction velocities. By training the PINNs on simulated data from the model, the framework achieves strong generalization to out-of-domain scenarios, such as complex scar patterns and multiple wavefronts (Kashtanova *et al*).

#### 3.1.6. Hodgkin–Huxley model

Since it was first introduced in 1952, the Hodgkin–Huxley model has been one of the most common PDEs formulated to describe how electrical signals (action potentials) are initiated and propagated along cells (Hodgkin and Huxley 1952). The model represents the cell membrane as an electrical circuit, where ionic currents flow through ion channels, driven by differences in ion concentrations and membrane voltage. The Hodgkin–Huxley model is considered the gold standard for biophysically detailed models, offering precise descriptions of ionic currents and their role in action potential generation.

The general form of the equations involved in the Hodgkin–Huxley model, as utilized in the PINNs, is expressed as,

\begin{align*} C \frac{\mathrm dV}{\mathrm dt} &amp; = I\left(t\right) - \sum_{i = 1}^{n} \overline{g}_{i} m^{k1} h^{k2} n^{k3} \left(V - E_i\right) - \overline{g}_L \left(V - E_L\right),\end{align*}

\begin{align*} \frac{\mathrm dm}{\mathrm dt} &amp; = \alpha_m\left(V\right)\left(1 - m\right) - \beta_m\left(V\right)m,\end{align*}

\begin{align*} \frac{\mathrm dn}{\mathrm dt} &amp; = \alpha_n\left(V\right)\left(1 - n\right) - \beta_n\left(V\right)n,\end{align*}

\begin{align*} \frac{\mathrm dh}{\mathrm dt} &amp; = \alpha_h\left(V\right)\left(1 - h\right) - \beta_h\left(V\right)h,\end{align*}

where *V* represents the membrane potential, *I*(*t*) is the external input current, *C* is the membrane capacitance, $\overline{g}_{i} $ and *E_i_* are the maximal conductance and reversal potential of the *i*th ion channel, $\overline{g}_L $ and *E_L_* are the conductance and reversal potential for the leakage current, $m, n, h $ are gating variables associated with ion channels, *α* and *β* functions are voltage-dependent rates for gating variables, $k1, k2, k3 $ are powers indicating the involvement of gating variables in the conductance formula. These equations simulate the dynamics of ion channels and contribute to understanding the overall EP behavior of the system under study.

The Hodgkin–Huxley model can explicitly be integrated into a PINNs framework and describe EP dynamics with a system of four ODEs. The PINNs framework solves these equations while estimating unknown parameters, making it suitable for both forward and inverse problems (Ferrante *et al* 2022). This framework can also be modified for estimating time-varying parameters in EP systems. The Hodgkin–Huxley model’s equations are augmented with time-varying conductances and gating parameters period, allowing the PINNs to dynamically predict these parameters while ensuring compliance with the Hodgkin–Huxley equations (Yao *et al* 2023).

#### 3.1.7. FitzHugh–Nagumo model

The FitzHugh–Nagumo model is a reduced and computationally efficient simplification of the Hodgkin–Huxley model, representing the dynamics of action potential generation and propagation. It reduces the biophysical complexity of the Hodgkin–Huxley model into two coupled equations: one for the membrane potential and another for a recovery variable,

\begin{align*} \frac{\mathrm dv}{\mathrm dt} = v - v^3 - w + R I_{\text{ext}}\end{align*}

\begin{align*} \frac{\mathrm dw}{\mathrm dt} = v + a - b w\end{align*}

where *v* is the membrane potential, *w* is the recovery variable, *R* is resistance, and *I*_ext_ is an external stimulus current. Parameters *a* and *b* modulate the recovery dynamics. This abstraction makes it suitable for PINNs applications, where interpretability and computational efficiency are prioritized (Rudi *et al* 2020, Ferrante *et al* 2022). Interpretability in this context refers to the model’s ability to transparently capture the core dynamical features of neuronal excitability (such as spike initiation, threshold behavior, and recovery) with only two variables. This facilitates a clear understanding of how inputs influence outputs, which aligns well with PINNs’ goal of learning physically meaningful relationships. Computational efficiency means that the FitzHugh–Nagumo model requires significantly fewer equations and parameters to solve, which reduces the training time and complexity of the PINNs. Because PINNs solve differential equations through neural network optimization, simpler models converge faster and are more tractable in high-dimensional or data-limited settings.

### 3.2. Muscle electromechanical models

Muscle electromechanical models consist of equations that integrate the electrical activity of muscles with their mechanical force generation. These models incorporate EP data, such as surface electromyography (sEMG) signals, with muscle mechanics based on force-generation models like the Hill-type or twitch-force equations. By connecting neural excitation to mechanical response, these models provide a more detailed and physiologically accurate understanding of muscle function. These models are particularly useful in fields such as biomechanics, rehabilitation, and prosthetics. In these areas, accurate muscle force predictions are crucial for designing and controlling assistive devices or understanding muscle behavior in health and disease (Petersen and Rostalski 2019). In the following, we briefly introduce two muscle electromechanical models that are commonly incorporated in PINNs.

#### 3.2.1. Hill-type model

The Hill-type musculoskeletal model describes the dynamics of muscle force generation. It includes two ODEs and few algebraic equations based on the concept of muscle force production, incorporating the force-length relationship, force-velocity relationship, and activation dynamics. In PINNs’ solution for muscle’s electromechanical modeling, these physics-based equations serve as constraints forcing the network’s predicted muscle forces to match the forces given by the Hill model (Taneja *et al* 2022, Zhang *et al* 2022, Ma *et al* 2024). The Hill-based muscle model for each musculotendon unit (MTU) force F can be given as (Buchanan *et al* 2004, Zhang *et al* 2022),

\begin{equation*} M\left(q\right) \ddot{q} + C\left(q, \dot{q}\right) + G\left(q\right) = \tau\left(t\right)\end{equation*}

\begin{equation*} T_a \frac{\mathrm da}{\mathrm dt} + a\left(t\right) = u\left(t\right)\end{equation*}

\begin{equation*} \tau\left(t\right) = \sum_{n = 1}^{N} F_{\text{mt}, n}\left(t\right) r_n\end{equation*}

\begin{equation*} F_{\text{mt}}\left(t\right) = \left[ F_{\text{max}}\,a\left(t\right)\,f_l\left(l_m\right)\,f_v\left(v_m\right) + F_{\text{max}}\,f_p\left(l_m\right) \right] \cos\left(\phi\right)\end{equation*}

Here, *q* denotes the joint angle, while $\dot{q}$ and $\ddot{q}$ are the joint angular velocity and acceleration, respectively. *M*(*q*) represents the mass (inertia) matrix as a function of joint angle, $C(q, \dot{q})$ is the Coriolis and centrifugal term, and *G*(*q*) is the gravitational torque. The variable $\tau(t)$ denotes the net joint torque. The muscle activation time constant is given by *T_a_*, *a*(*t*) is the muscle activation, and *u*(*t*) is the neural excitation (input, for example, from EMG). The model considers *N* muscles crossing the joint, where $F_{\text{mt}, n}(t)$ is the muscle-tendon force generated by the *n-*th muscle and *r_n_* is its corresponding moment arm. $F_{\text{mt}}(t)$ denotes the muscle-tendon force, *F*_max_ is the maximum isometric force, $f_l(l_m)$ is the normalized force-length relationship as a function of muscle fiber length *l_m_*, $f_v(v_m)$ is the normalized force-velocity relationship as a function of muscle contraction velocity *v_m_*, $f_p(l_m)$ is the normalized passive force-length relationship, and *φ* is the pennation angle between muscle fibers and the tendon.

#### 3.2.2. Twitch force model

The twitch force model mathematically describes how the force of a single motor unit (MU) is related to its electrical activation in response to neural stimulation. The model typically uses a second-order ODE to describe the force-time relationship for each MU, although its closed-form solution is often used when incorporated into a PINNs framework. In the full muscle model, the twitches of all MUs sum to produce the total muscle force, and sEMG signal reflects the combined electrical activity from multiple motor units within a muscle, captured at the surface of the skin (Li *et al* 2022). The closed-form of twitch force model can be expressed as,

\begin{equation*} f_i\left(t\right) = \frac{P_i t}{T_i}\mathrm e^{1-\frac{1}{T_i}}\end{equation*}

which is the closed-form solution of

\begin{align*} \frac{\mathrm d^2 f\left(t\right)}{\mathrm dt^2} + \frac{2}{T_i} \frac{\mathrm df\left(t\right)}{\mathrm dt} + \frac{1}{T_i^2} f\left(t\right) = 0\end{align*}

with the initial conditions of

\begin{align*} f\left(0\right) = 0,\quad \left.\frac{\mathrm df}{\mathrm dt}\right|_{t = 0} = \frac{P_i}{T_i}\end{align*}

where *T_i_* is the contraction time of the twitch force, *P_i_* is the amplitude of twitch force and *i* is the MU index in the pool containing all MUs.

### 3.3. Hemodynamic models

Hemodynamic models describe the dynamics of blood flow within the cardiovascular system, governed primarily by the principles of fluid mechanics, such as mass and momentum conservation. These models often simulate pressure and velocity fields within blood vessels, enabling the assessment of critical metrics like blood pressure, fractional flow reserve (FFR) or wall shear stress (WSS). In the context of PINNs, hemodynamic models are directly embedded into the learning process through the loss function. By penalizing deviations from the governing ODEs/PDEs, PINNs integrate physical constraints into data-driven models. This hybrid approach allows PINNs to learn accurate flow patterns even from sparse or noisy data while ensuring physiologically consistent outputs, making them suitable for non-invasive cardiovascular diagnostics and patient-specific simulations. This section discusses some of the most widely used models in hemodynamic PINNs.

#### 3.3.1. Navier–Stokes equations

The Navier–Stokes equations govern the flow of incompressible fluids by balancing internal fluid stresses with external forces. This model is the most essential for simulating fluid flow dynamics in complex geometries (Jin *et al* 2021). The blood flow in a vessel can also be modeled using the Navier–Stokes equations,

\begin{align*} &amp; \frac{\partial A}{\partial t} + \frac{\partial Q}{\partial z} = 0\end{align*}

\begin{align*} &amp; \frac{\partial Q}{\partial t} + \frac{\partial}{\partial z} \left( \frac{Q^2}{A} \right) + \frac{A}{\rho} \frac{\partial P}{\partial z} + \frac{K_r Q}{A} = 0\end{align*}

\begin{align*} &amp; P = P_{\text{ext}} + \beta \left( \sqrt{A} - \sqrt{A_0} \right)/A_0 \quad\end{align*}

where *A* is the cross-sectional area, *Q* is the blood flow rate, *z* is the spatial coordinate, *P* is the blood pressure, *ρ* is the blood density, *K_r_* is a resistance parameter, *P*_ext_ is the external pressure, *A*_0_ is the reference cross-sectional area, *β* is a constant.

Navier–Stokes equations have been utilized to understand blood flow in various cardiovascular conditions using a PINNs approach. This includes advanced boundary conditions such as Windkessel models to simulate realistic cardiovascular dynamics (Li *et al* 2024). In another study, other additional conditions like continuity of velocity and pressure field have been employed beside the Navier–Stokes equations to improve the accuracy (Du *et al* 2023).

In a recent study, the Navier–Stokes equations were employed to model mass transport and diffusion in simulations of blood flow through arterial bifurcations, with a focus on parameter estimation and on ensuring mass conservation and pressure continuity across the domain (Isaev *et al* 2024b).

In addition, this model has been used to examine Fourier-based activation functions in PINNs to model patient-specific cardiovascular flows, highlighting their ability to capture the high-frequency dynamics typical in cardiovascular diseases such as aneurysms or stenoses (Aghaee and Khan 2024).

The Navier–Stokes model can be applied within a PINNs framework to address the vector flow mapping (VFM) problem, using color-Doppler sequences and time-resolved delineations of the left ventricular (LV) endocardial wall as inputs. The framework outputs a phase-unwrapped color-Doppler map, reconstructed cross-beam velocity, and fluctuating pressure maps. The PINNs are trained on patient-specific data by minimizing a loss function that combines the differences between the input and output Doppler maps with the residuals of the continuity and Navier–Stokes equations, including boundary conditions at the endocardial border (Maidu *et al* 2025).

Navier–Stokes equations have been also utilized as the required PDEs in PINNs to estimate near-wall blood flow and WSS, in 1-D advection-diffusion transport, 2D blood flow in a stenosis, 2D blood flow in an aneurysm, and 3D blood flow in an idealized aneurysm (Arzani *et al* 2021, Moser *et al* 2023, Sautory and Shadden 2024).

Some other studies employ a one-dimensional model of pulsatile flow, which is a simplification of the Navier–Stokes equations in addition to Laplace’s law, to predict arterial blood pressure from 4D flow MRI data (Kissas *et al* 2020).

#### 3.3.2. Linearized Navier–Stokes equations

The Navier–Stokes equations govern fluid motion and, in cylindrical coordinates for incompressible Newtonian flow, they are quite complex. For blood flow in arteries, these equations can be simplified through several physiologically justified assumptions. First, it is assumed that blood vessels are sufficiently thin and approximately straight over short segments, allowing the equations to be reduced to one dimension. Second, small perturbations around a steady state are considered negligible, which permits the linearization of the equations (Liang *et al* 2023),

\begin{align*} k_p \frac{\partial P}{\partial t} + A_0 \frac{\partial u}{\partial x} &amp; = 0\end{align*}

\begin{align*} \frac{\partial u}{\partial t} + \frac{1}{\rho} \frac{\partial P}{\partial x} + K_u u &amp; = 0\end{align*}

where *P* is pressure, *u* is velocity, *x* is axial position, *t* is time, *A*_0_ is baseline cross-sectional area, *ρ* is blood density, *k_p_* is compliance, and *K_u_* is the damping coefficient.

This reduced model can play a central role to solve the pulse wave inverse problem (PWIP) in the field of pulse wave imaging (PWI). PWI is a noninvasive ultrasound-based technique for evaluating arterial compliance and pulse wave velocity (PWV). PWV is closely linked to arterial wall mechanics and is often interpreted through the Bramwell-Hill model (Rabben *et al* 2004). When combined with vector flow imaging (VFI), PWI enables high-resolution characterization of hemodynamic patterns and arterial properties. In PWIP solving, the linearized Navier–Stokes equations can be integrated in PINNs to facilitate the estimation of vascular mechanical parameters and hemodynamic conditions (Liang *et al* 2023).

#### 3.3.3. Burger equations

A viscoelastic model of arterial flow derived from the Burger equations and Korteweg–de Vries (KdV) can be utilized to simulate pressure and radius perturbations in arterial blood flow, considering nonlinear elasticity and viscoelasticity of the walls of the tube (Bhaumik *et al* 2024). The Burger equation is given as,

\begin{equation*} \frac{\partial u}{\partial t} + u\frac{\partial u}{\partial x} = \frac{1}{2}\frac{\partial^2u}{\partial x^2}\end{equation*}

where *u* represents the velocity profile at different scales of time, *t* is the time variable, *x* is the spatial variable.

### 3.4. Other fields

In addition to physiological signals, PINNs are utilized in the analysis of various types of biomedical data, including heat distribution and thermal modeling, particularly in cancer diagnostics or tissue temperature regulation (Ottens *et al* 2022, Mukhmetov *et al* 2023, Perez–Raya and Kandlikar 2023). Simulations of tissue mechanics, such as cardiac mechanics or skeletal muscle behavior, utilizing mechanical laws and finite element methods (FDMs) is also another field for which PINNs with proper PDEs can be beneficial (Buoso *et al* 2021, Alzhanov *et al* 2024, Motivale *et al* 2024).

In addition, there are studies in the field of physiological signal processing that utilize physics models (such as algebraic models or domain boundary sets) alongside neural networks, but do not incorporate these models into the loss function. Notable examples include work in EEG source localization (Morik *et al* 2024, Waters and Clifford 2024) and brain-computer interfacing (Altaheri *et al* 2023). However, some of these studies adopt the title of PINNs, while others do not, though none of them fit the classic definition of PINNs. To avoid ambiguity in the present review, we stick to the classic definition and focus primarily on studies that employ PDEs or ODEs as the governing physics models in the loss function of their PINN formulations.

Tables 4 and 5 give a summary of PDE models’ properties and applications in PINNs analysis of physiological signals in detail. The PDE models adopted in electrophysiology, muscle electromechanics, and hemodynamics are explained in detail. Table 4 summarizes the number of ODEs/PDEs adopted in each model, the number of variables, the dimensionality of the domain, and the boundary conditions of the ODEs/PDEs. Table 5 summarizes the primary implementation of the models, the types of problems they address, the types of data used to train them, and the outputs of the ODEs/PDEs. As indicated in table 5, in most cases, the ODEs/PDEs are used to solve forward problems in physiological signal processing and modeling. As mentioned in section 2, multiple terms are used to construct the loss function, with the ODEs/PDEs incorporated as residual terms within it.

**Table 4.** PDE models’ properties in PINNs analysis of physiological signals.

Field	Model	No. of PDE/ODE	No. of Vars	Domain Dimension	Boundary conditions
Electrophysiology	Eikonal (Sahli *et al* 2020, Grandits *et al* 2021, Jiang *et al* 2024)	1 PDE	2 & 3	2D & 3D	N/A
	State-Space (Jiang *et al* 2024)	Neural ODEs	64	3D	N/A
	Aliev–Panfilov (Herrero *et al* 2022, Xie and Yao 2022a, 2022b, Chiu *et al* 2024)	1 ODE + 1 PDE	4	1-D & 2D & 3D	Neumman
	Fenton–Karma (Sahli *et al* 2020, Chiu *et al* 2024)	2 ODEs + 1 PDE	4	2D & 3D	Neumman
	Mitchell–Schaeffer (Kashtanova *et al* 2021, Kashtanova *et al* 2022a)	2 ODEs	3 & 4	2D & 3D	N/A
	Hodgkin–Huxley (Ferrante *et al* 2022, Yao *et al* 2023)	4 ODEs	5	1-D	N/A
	FitzHugh–Nagumo (Rudi *et al* 2020, Ferrante *et al* 2022)	2 ODEs	3	1-D	N/A
Muscle Electro- mechanics	Hill-type model (Taneja *et al* 2022, Zhang *et al* 2022, Ma *et al* 2024)	2 ODEs	3	1-D	N/A
	Twitch force model (Li *et al* 2022)	1 ODE	no. of MUs	1-D	N/A
Hemodynamics	Navier–Stokes + Windkessel (Li *et al* 2024)	2 PDEs	3	1-D	Windkessel
	Navier–Stokes + continuity (Arzani 2021, Du *et al* 2023, Moser 2023, Isaev *et al* 2024b, Sautory and Shadden 2024, Maidu *et al* 2025)	3 PDEs	8	1-D & 2D & 3D	Dirichlet & Neumman
	Navier–Stokes + Laplace (Kissas *et al* 2020)	2 PDEs	5	4D	Windkessel
	Burger + KdV (Bhaumik *et al* 2024)	2 PDEs	3	1-D	Neumman
	Linearized Navier–Stokes (Liang *et al* 2023)	2 PDEs	4	1-D	Dirichlet

‘N/A’ indicates that either no boundary condition is applied in the model or the paper does not explicitly provide information about the boundary condition.

**Table 5.** PDE models’ applications in PINNs analysis of physiological signals.

Field	Model	Focus	Type of problem	Type of data	Output
Electrophysiology	Eikonal (Sahli *et al* 2020, Grandits *et al* 2021, Jiang *et al* 2024)	Spatial mapping	Forward	*In silico* data	Activation maps
	State-Space (Jiang *et al* 2024)	Spatial mapping	Forward	*In silico* data; *In vivo* data	ECGI
	Aliev–Panfilov (Herrero *et al* 2022, Xie and Yao 2022a, 2022b, Chiu *et al* 2024)	Spatial-temporal evolution	Forward; Inverse; Forward & Inverse	*In silico* data	Activation maps + ECGI
	Fenton–Karma (Sahli *et al* 2020, Chiu *et al* 2024)	Spatial-temporal evolution	Inverse;Forward & Inverse	*In silico* data	Activation maps
	Mitchell–Schaeffer (Kashtanova *et al* 2021, Kashtanova *et al* 2022a)	Spatial-temporal evolution	Forward; Forward & Inverse	*In silico* data	Activation maps
	Hodgkin–Huxley (Ferrante *et al* 2022, Yao *et al* 2023)	Time series prediction	Inverse	*In silico* data	Neuron action potential
	FitzHugh–Nagumo (Rudi *et al* 2020, Ferrante *et al* 2022)	Time series prediction	Inverse	*In silico* data	Neuron action potential
Muscle Electro- mechanics	Hill-type model (Taneja *et al* 2022, Zhang *et al* 2022, Ma *et al* 2024)	Time series prediction	Forward	*In vivo* data	Muscle force
	Twitch force model (Li *et al* 2022)	Time series prediction	Forward	*In vivo* data	Muscle force
Hemodynamics	Navier–Stokes + Windkessel (Li *et al* 2024)	Time series prediction	Forward & Inverse	*In vivo* data	Blood pressure
	Navier–Stokes + continuity (Arzani 2021, Du *et al* 2023, Moser 2023, Isaev *et al* 2024b, Sautory and Shadden 2024, Maidu *et al* 2025)	Spatial mapping	Forward	*In silico* data	Blood pressure
	Navier–Stokes + Laplace (Kissas *et al* 2020)	Time series prediction	Forward	*In vivo* data	Blood pressure
	Burger + KdV (Bhaumik *et al* 2024)	Spatial-temporal evolution	Forward	*In silico* data	Blood pressure + velocity
	Linearized Navier–Stokes (Liang *et al* 2023)	Spatial-temporal evolution	Inverse	*In silico* data	Blood pressure

### 3.5. Conventional numerical methods

Traditionally, ODEs/PDEs in physiological modeling have been solved using established numerical techniques such as finite difference methods (FDMs), finite element methods (FEMs), and finite volume methods (FVMs). These methods have been successfully employed in modeling cardiac electrophysiology (Nash and Panfilov 2004), blood flow dynamics (Quarteroni *et al* 2017), and muscle electromechanics (Kojic *et al* 2019). They offer high numerical accuracy and a well-understood mathematical foundation. However, they often require problem-specific spatial discretization, mesh generation, and can be computationally expensive, particularly in three-dimensional domains or when simulating coupled multiphysics phenomena (Lo *et al* 2024). Moreover, incorporating patient-specific data or solving inverse problems using these traditional solvers typically demands substantial preprocessing and manual tuning.

PINNs address several of these limitations. As mesh-free solvers, PINNs can be applied directly to irregular geometries and sparse datasets. They encode the governing physical laws in the loss function, enabling simultaneous learning from data and physics (Raissi *et al* 2019). This makes them especially advantageous for inverse problems and parameter estimation in physiological models. Nonetheless, PINNs come with their own challenges, including optimization difficulties, sensitivity to loss term weighting, and scalability to high-dimensional problems (Cuomo *et al* 2022).

## 4. Overview of characteristics and metrics

The limitations of this review paper lie in its focus on studies published within the past five years (2019–2024) related to physiological modeling, excluding areas such as medical image processing. The signal characteristics and machine learning metrics describing the results of PINNs for various areas are summarized in multiple tables. Table 6 summarizes the PINNs results in cardiac electrophysiology, the objective, signal characteristics, and machine learning metrics adopted in each paper are listed in detail. The items are ranked based on the year of publication. Table 7 summarizes the results for PINNs in the areas of neural dynamics, cancer, and electromyography. Items 1 to 2 summarize the results for neural dynamics. Items 3 to 5 summarize the results for cancer. Items 6 to 8 summarize the results for electromyography. Tables 8 and 9 summarize the results of PINNs in hemodynamics. Most of the papers in cardiac electrophysiology focus on cardiac electrophysiology modeling and dynamics forecasting, typically demonstrating high accuracy in testing and evaluation. This suggests that significant efforts have been directed toward accurately capturing the electrical activity of the heart, which plays a critical role in understanding arrhythmias and other cardiac conditions, such as diastolic heart failure. For hemodynamic studies, most of the research focuses on the prediction of blood flow, covering various aspects such as blood flow modeling, parameter estimation, and reconstruction. These studies aim to improve the understanding of hemodynamic patterns, which are critical for diagnosing and managing cardiovascular diseases. To assess the performance of these models, multiple machine learning metrics are employed, including RE, MAE, MSE, RMSE, and NRMSE. Each of these metrics provides different insights into the model’s accuracy and reliability. The adoption of multiple evaluation criteria ensures a comprehensive assessment of model performance, helping researchers identify strengths and limitations in predictive accuracy. Moving forward, incorporating domain-specific constraints and enhancing model interpretability will be essential for advancing various biomedical fields, including cardiac electrophysiology, hemodynamics, neurodynamics, cancer research, and musculoskeletal studies.

**Table 6.** Summary of the results for PINNs in cardiac electrophysiology.

Article	Objective	Signal characteristics	Error metrics
Sahli *et al* (2020)	Cardiac activation mapping	*In silico* data	RMSE
Grandits *et al* (2021)	Estimate heart conductivity tensor	*In silico data;* *In vivo* electroanatomical maps (EAMs)	RMSE
Herrero *et al* (2022)	Electrophysiological tissue parameters estimation	*In silico* data; *In vitro* data	RMSE
Xie and Yao (2022a, 2022b)	Spatiotemporal cardiac electrodynamics modeling	*In silico* data	RE
Tenderini *et al* (2022)	Epicardial potentials and activation maps estimation	*In silico* data	Relative L1-norm error
Kashtanova *et al* (2022b)	Cardiac electrophysiology dynamics forecasting	*In silico* data; *Ex vivo* optical mapping data	MSE
Kashtanova *et al* (2023)	Cardiac electrophysiology dynamics forecasting	*Ex vivo* data	MSE
Yao *et al* (2023)	Ion channels dynamics prediction	*In silico* data; Real measure membrane potential dataset	RMSE; Pearson coefficient
Nazaret *et al* (2023)	Heart rate (HR) response prediction	*In vivo* ECG data	MAE
Xie (2023)	Inverse ECG modeling	*In silico data;* Body surface potential mapping (BSPM) data	MSE; RE; Correlation coefficient (CC)
Chiu *et al* (2024)	Drug effects on cardiac electrophysiology	*In vitro* optimal mapping images	RMSE; RE
Chiu *et al* (2024)	Cardiac electrophysiology dynamics forecasting with complex geometries and dynamic regimes	*In silico* data	RMSE
Jiang *et al* (2024)	Electrocardiographic imaging	*In silico* data; *In vivo* data	MSE
Jiang *et al* (2024)	Cardiac electrophysiology dynamics forecasting	*In silico* data	MSE
Kuang *et al* (2024)	Create patient-specific digital twins using non-invasive echocardiography data	*In silico* pressure-volume data; *In vivo* echocardiography	MAE
Motiwale *et al* (2024)	High speed cardiac mechanics simulations	Random *in silico* data in space	ME

Definitions of RMSE, MSE, MAE, ME and RE can be found in table 2.

**Table 7.** Summary of the results for PINNs in the areas of neural dynamics, cancer, and electromyography.

Article	Objective	Signal characteristics	Error metrics
Sun *et al* (2022)	Neural mass model	*In silico* data	Pearson correlation; Average localization error
Ferrante *et al* (2022)	Neural modeling	*In silico* data	MSE
Ottens *et al* (2022)	Pancreatic cancer	*In silico* data; Clinical dataset	NRMSE
Mukhmetov *et al* (2023)	Temperature prediction in cancerous breasts	*In silico* data	Relative *L*_1_-norm error
Perez-Raya and Kandlikar (2023)	Thermal modeling of breast cancer	*In silico* data	RE
Taneja *et al* (2022)	Motion prediction and parameter identification of human MSK systems	Surface electromyography data	MSE
Li *et al* (2022)	Muscle force decoding	Surface electromyography data	RMSE
Zhang *et al* (2022)	Ankle joint torque prediction	Surface electromyography data	NRMSE
Ma *et al* (2024)	Muscle force prediction	Surface electromyography data	RMSE

Definitions of NRMSE, RMSE, MSE, and RE can be found in table 2.

**Table 8.** Summary of the results for PINNs in hemodynamics.

Article	Objective	Signal characteristics	Error metrics
Kissas *et al* (2020)	Arterial blood flow prediction	*In vivo* data	Relative *L*_2_ Error
Buoso *et al* (2021)	Ventricular biomechanics simulation	*In silico* data	RE
Sarbian *et al* (2022)	Brain hemodynamic prediction	*In vivo* data	Maximum relative *L*_2_ error
Du *et al* (2023)	Aortic hemodynamics	*In silico* data	RE
Liang *et al* (2023)	Noninvasive intravascular pressure estimate	*In silico* data	MSE;RE
Moser *et al* (2023)	3D blood flow modeling	*In silico* data	MAE
Wang *et al* (2024)	Cuffless blood pressure estimation	Clinical blood pressure datasets	RMSE
Bhaumik *et al* (2024)	Arterial blood flow wave process	*In silico* data	MSE
Liu *et al* (2024)	Prediction of fractional flow reserve	Clinical FFR data	MSE; Coefficient of determination
Li *et al* (2024)	Blood flow continuously prediction	*In silico* data;MIMIC dataset	RE; MAE
Maidu *et al* (2025)	Ventricular flow and pressure fields prediction	*In silico* flow data	NRMSE

Definitions of NRMSE, RMSE, MSE, MAE, and RE can be found in table 2.

**Table 9.** Summary of the results for PINNs in hemodynamics.

Article	Objective	Signal characteristics	Error metrics
Aghaee and Khan (2024)	Cardiovascular blood flows modeling	*In silico* data	*L*_2_ norm Squared error
Isaev *et al* (2024a)	Arterial blood flow parameter estimation	*In silico* data	RE
Isaev *et al* (2024b)	Blood flow parameter estimation	*In silico* data	Relative MSE
Alzhanov *et al* (2024)	Blood flow prediction	*In silico* data	Mean RE
Arzani *et al* (2021)	Wall shear stress quantification in diseased arterial flows	*In silico* data	*L*_2_ norm
Sautory and Shadden (2024)	Blood flow reconstruction	*In silico* data	MSE;RMSE; CC

Definitions of RMSE, MSE, and RE can be found in table 2.

## 5. Discussion

In this review paper, the application of PINNs in the processing and modeling of physiological signals is explored in detail. An increasing trend has been observed in the use of PINNs in various fields, particularly in cardiac electrophysiology and hemodynamics. PINNs have been applied to cardiovascular flow prediction, cardiac electrophysiology dynamics forecasting, and blood flow parameters estimation, serving various purposes in cardiac electrophysiology modeling. There are also few applications in neural dynamics prediction, cancer diagnostics, and electromyography modeling. The most obvious strength of PINNs is that it provides an opportunity to incorporate the physics constraints with the data-driven neural networks algorithms. PINNs require fewer data points and labels to learn compared to a traditional data-driven approach. Moreover, the integration structure of PINNs allows one to use a simplified low-fidelity physics model, which reduces the requirements for the modeling and computational cost. At the same time, incorporating physics models into the boundary conditions of neural networks can help ensure that scientific principles are embedded in the predictions. It can help PINNs generalize better to unseen scenarios, and more robust to noisy or incomplete biomedical data with the help of physics-based constraints. Instead of only solving the PDEs, model parameters can also be adopted as trainable weights in the construction of PINNs. This provides the opportunity to develop patient-specific neural networks model with given datasets. Moreover, the PINNs provides better interpretability for neural networks. PINNs can be treated as a mesh-free approach to avoid the cumbersome processes involved in traditional computation algorithms like finite element analysis (FEA) or CFD.

In terms of the limitations of PINNs, the primary concern lies in the optimization approach. Given the numerous possible choices for network structure and hyperparameters, identifying optimal configurations is crucial for improving both the performance and reproducibility of PINNs. A notable study addressing optimization in PINNs, particularly the automatic selection of weighting parameters for the loss function, is presented in de Wolff *et al* (2021). The computational cost is another important consideration, particularly when working with patient-specific models, as training for each patient can be time-consuming. In such cases, transfer learning can be employed to reduce training time by leveraging a pre-trained neural network model. Another limitation of PINNs is the local minimum issue, which is often observed in the training process of machine learning algorithms. To overcome the local minimum issue, more advanced optimization algorithms and neural networks architectures are required. Adam is the most commonly used optimizer for PINNs and the L-BFGS optimizer (Liu and Nocedal 1989) can help overcome local minima issues, but they depend heavily on the initialization. L-BFGS is an improved version of the BFGS optimization algorithm that uses a limited-memory approach to approximate the Hessian matrix for large-scale optimization. Combining Adam and L-BFGS is a common strategy that leverages the strengths of first-order and second-order optimization methods to achieve faster and more accurate convergence, while also helping to overcome ill-conditioning issues in minimizing the PINNs loss function (Rathore *et al* 2024). Moreover, it is also important to emphasize the scalable and reliable methods for uncertainty quantification in PINNs (Kissas *et al* 2020). It has been investigated that uncertainty quantification can be solved using the randomized prior functions in Sahli *et al* (2020). PINNs also show limitations in terms of the curse of dimensionality and spectral bias during training convergence. The curse of dimensionality is a major challenge in solving PDEs, and PINNs also fail to address it due to insufficient memory and slow convergence when handling high-dimensional PDEs (Hu *et al* 2024). In terms of spectral bias, PINNs show various convergence rates for high-frequency and low-frequency features. PINNs often struggle to converge when PDEs have high-frequency features (Farhani *et al* 2022). PINNs have also been investigated to suffer from a discrepancy of convergence rate in the different components of their loss function (Wang *et al* 2022). Except for the limitations for the inherent properties of PINNs, PINNs also show challenges when employed for physiological signal processing and modeling. Although various PINNs structures have been investigated in these applications, *in silico* data is commonly used to evaluate model performance. The limitations of these models in clinical validation deserve further investigation. Clinical data often contains noise, artifacts, and sparsity, posing significant challenges for PINNs predictions. Data collection and preprocessing techniques need further investigations before implementing the PINNs structure directly. Bias is another critical factor in physiological signal modeling. However, most of the reviewed papers overlook the impact of bias in the modeling of PINNs. For example, demographic information impacts the generalization of the PINNs model due to the inherent variability in human physiology across different population groups and needs to be considered during the modeling.

There is an emerging trend in applying PINNs in physiological signal processing and modeling. Except for the limitations mentioned above, the potential of PINNs to serve as a patient-specific robust architecture for physiological signal modeling remains an open and critical problem to address. Patient-specific models can be built with the help of fine-tuning and transfer learning, however, real-time analysis remains challenging due to the computational burden of implementing neural networks on bedside medical devices. In most cases, PINNs are integrated with specific nonlinear PDEs to include the physical constraints, allowing simultaneous implementation of parameter estimation. However, there are few investigations into scenarios where PDEs are too complex to model directly, such as multiscale dynamic systems, nonlinear and coupled systems, and systems with complex initial and boundary conditions. In such cases, data-driven algorithms can assist in approximating these PDEs.

## 6. Conclusion

This review paper aims to summarize the applications of PINNs in physiological signal processing and modeling, covering commonly used physics models, neural network architectures, loss functions, input data modalities, as well as strengths and limitations of PINNs. PINNs perform well in physiological signal processing and modeling across various domains. Nevertheless, significant challenges remain concerning computational costs, input data preprocessing, and performance improvements necessary for developing robust PINNs models. Further investigations into the PINNs framework are required to enhance its performance. For example, integrating PINNs with trending AI models, such as transformers, represents another promising direction. Moreover, comparing the performance of PINNs and purely data-driven algorithms is a valuable area for further investigation. Understanding how to select between PINNs and data-driven approaches based on different conditions is particularly interesting, such as identifying scenarios where PINNs excel and where data-driven algorithms may be more effective. The reviewed studies highlight the strong potential of PINNs as a dependable, precise, and efficient tool for tackling complex problems in physiological signal processing and modeling.

## Data Availability

There is no research data being used in this paper. The data that support the findings of this study are available upon reasonable request from the authors.
